# Trends in health literacy discussions within primary health care research: A topic analysis using machine learning techniques

**DOI:** 10.1016/j.aprim.2025.103441

**Published:** 2026-01-15

**Authors:** Muhammet Damar, Andrew David Pinto, Benita Hosseini, Thiago Gomes da Trindade, Ömer Aydın, Fatih Safa Erenay, Ümit Cali

**Affiliations:** a Management Information Systems, Faculty of Business Administration, Muğla Sıtkı Koçman University, Muğla, Turkey; b Upstream Lab, MAP, Li Ka Shing Knowledge Institute, Unity Health Toronto, Toronto, Ontario, Canada; c Department of Family and Community Medicine, Faculty of Medicine, University of Toronto, Toronto, Ontario, Canada; d Dalla Lana School of Public Health, University of Toronto, Toronto, Ontario, Canada; e Department of Family and Community Medicine, St. Michael's Hospital, Unity Health Toronto, Toronto, Ontario, Canada; f Department of Clinical Medicine, Federal University of Rio Grande do Norte, Brazil; g Computer Science, Electrical and Electronics Engineering, Faculty of Engineering and Natural Sciences, Manisa Celal Bayar University, Manisa, Turkey; h Department of Management Sciences, University of Waterloo, Waterloo, Ontario, Canada; i Management Science and Engineering, Faculty of Engineering, University of Waterloo, Waterloo, Ontario, Canada; j Department of Electric Power Engineering, Faculty of Information Technology and Electrical Engineering, Norwegian University of Science and Technology, Trondheim, Norway; k School of Physics, Engineering and Technology, York University, England, United Kingdom

**Keywords:** Health literacy, Primary care, Health care, Text mining, Machine learning, Alfabetización en salud, Atención primaria, Atención médica, Minería de textos, Aprendizaje automático

## Abstract

**Objective:**

To reveal the intellectual framework, research trends, and gaps, and evaluate effective health literacy tools in the field of primary healthcare services.

**Design:**

Observational, machine learning-based bibliometric study.

**Site:**

Analysis was conducted using records indexed in the Web of Science database.

**Participants:**

A total of 1869 researchers from 823 institutions across 54 countries contributed to the 33 journals included in the dataset.

**Interventions:**

Development of a bibliometric map and topic model in health literacy within primary healthcare. Bibliometric analysis was performed on review and research articles retrieved as of July 27, 2025. For each article, data on the journal, publication year, title, abstract, keywords, authors, affiliations, countries, cited sources, cited first authors, and references were collected. Latent Dirichlet Allocation topic modeling was applied to uncover thematic structures and trends in the research field.

**Main measurements:**

Thematic structures, research trends, and knowledge gaps were measured through bibliometric indicators such as co-authorship networks, citation analysis, and topic modeling outputs.

**Results:**

Emerging topics included health equity and sustainability, medication adherence, aging, management of lifestyle factors such as physical activity and diet, management of chronic diseases, physician-patient communication, sustainable learning, sociodemographic impact, rural health interventions, responses to pandemics akin to COVID-19, and the roles of health institutions, policymakers, and leadership.

**Conclusions:**

Our findings highlight that health literacy is a multifaceted concept that not only enables healthier living and disease management but also prevents various severe health conditions, improving overall life quality and satisfaction with health services. The importance of sustained health literacy initiatives, effective communication, and the vested interests of both patients and healthcare professionals are highlighted, underscoring the need for ongoing commitment in this area.

## Abstract (es)

**Objetivos:**

Revelar el marco intelectual, las tendencias y las lagunas en la investigación, y evaluar las herramientas eficaces de alfabetización en salud en el ámbito de los servicios de atención primaria de salud.

**Diseno:**

Estudio observacional bibliométrico basado en el aprendizaje automático.

**Ámbito:**

El análisis se llevó a cabo utilizando registros indexados en la base de datos Web of Science.

**Participantes:**

Un total de 1.869 investigadores de 823 instituciones en 54 países contribuyeron a las 33 revistas incluidas en el conjunto de datos.

**Intervenciones:**

Desarrollo de un mapa bibliométrico y modelo temático en alfabetización en salud en la atención primaria de salud. Se realizó un análisis bibliométrico de artículos de revisión e investigación recuperados hasta el 27 de julio de 2025. Para cada artículo se recopilaron datos sobre la revista, año de publicación, título, resumen, palabras clave, autores, afiliaciones, países, fuentes citadas, primeros autores citados y referencias. Se aplicó el modelado de temas Latent Dirichlet Allocation para identificar las estructuras temáticas y las tendencias en el campo de investigación.

**Mediciones principales:**

Las estructuras temáticas, las tendencias de investigación y las brechas de conocimiento se midieron mediante indicadores bibliométricos como redes de coautoría, análisis de citas y resultados de modelado de temas.

**Resultados:**

Entre los temas emergentes se incluyeron la equidad y la sostenibilidad en el ámbito de la salud, la adherencia al tratamiento farmacológico, el envejecimiento, la gestión de factores relacionados con el estilo de vida, como la actividad física y la alimentación, el manejo de enfermedades crónicas, la comunicación entre médicos y pacientes, el aprendizaje sostenible, el impacto sociodemográfico, las intervenciones sanitarias en zonas rurales, las respuestas a pandemias similares a la COVID-19 y el papel de las instituciones sanitarias, los formuladores de políticas públicas y los líderes.

**Conclusiones:**

Nuestros hallazgos destacan que la alfabetización en salud es un concepto multifacético que no solo permite llevar una vida más saludable y el manejo de enfermedades, sino que también previene diversas afecciones graves, mejorando la calidad de vida general y la satisfacción con los servicios sanitarios. Se destaca la importancia de las iniciativas sostenidas de alfabetización en salud, la comunicación eficaz y de la consideración de los intereses tanto de los pacientes como de los profesionales de salud, lo que evidencia la necesidad de un compromiso continuo en este ámbito.

## Introduction

Health literacy encompasses skills and knowledge related to health and healthcare, enabling individuals to locate, understand, interpret, and communicate health information, to seek appropriate care. Numerous definitions of health literacy exist. Luckenbaugh and Moses^12^ define health literacy as the ability to access, understand, and act upon medical information to make health-related decisions. Gallè et al.^13^ describe health literacy as a fundamental skill for managing health and disease issues. Konopik et al.^14^ define health literacy as a lifelong competency relevant to age-related health limitations, while Sariyar and Firat Kiliç^15^ express health literacy as the motivation and ability to acquire, understand, evaluate, and utilize health information.

Improved health literacy helps individuals to make more informed medical decisions, and thus, better manage their health and adopt healthy behaviors.^1^, ^2^, ^7^, ^8^ In addition, effective communication between patients and healthcare providers relies heavily on patient health literacy and understanding of medical terminology, which also improves adherence to medication guidelines and plans.^6^, ^7^ Conversely, low health literacy leads to inefficient use and delivery of healthcare, negatively impacting health outcomes and healthcare costs.^9^

Health literacy has been acknowledged as a critical determinant of health in the 2016 Shanghai Declaration.^11^ The role of health literacy in reducing health disparities and improving the efficiency of basic health services is increasingly recognized.^10^ Limited health literacy is increasingly recognized as a significant barrier to receiving adequate healthcare, and is associated with poor patient health outcomes and increased hospitalization rates.^4^, ^5^ However, still half of adults lack the necessary health literacy skills for navigating complex health care systems.^3^ The effective dissemination of health information and improvement of health literacy can reduce barriers to healthcare access in underserved communities and help mitigate health inequalities.^16^ Previous studies demonstrated a positive relationship between health literacy and health knowledge, behaviors, and status.^17^ Adequate health literacy is crucial for clear communication between patients and healthcare providers.^18^ Health literacy can be faciliated through written, oral, or digital formats.^19^

Research on health literacy is expanding globally. The impact of literacy on health and healthcare remains a significant research area.^20^ Our study aims to analyze scientific research and review articles related to health literacy in the field of primary health care research in depth for all years using bibliometric methods and the machine learning technique of Latent Dirichlet Allocation (LDA) topic analysis. In this way, current topics, topic clusters, and highly cited topics in the health literacy literature conducted in the primary health care research field can be evaluated. In addition, the analysis provides information on prominent researchers, institutions, countries, journals, references, and citations.

## Materials and methods

Creating a systematic evaluation in a specific research domain and providing a comprehensive assessment for traditional literature analysis can be challenging due to the abundance of research in the field. Bibliometric methods enable access to a wealth of information about research outputs in a particular area. Additionally, the LDA method allows for topic modeling, organizing high-content text documents, summarizing them, and deriving valuable insights and assessments. In this study, both methods were utilized.

Specifically, this study systematically analyzes 393 bibliometric records comprising research and review articles in the field of health literacy in primary care research, determining annual publication outputs, leading countries, articles, journals, institutions, and prominent topic headings. Moreover, articles in the field of health literacy are systematically analyzed through bibliometric methods and the LDA topic analysis method to evaluate their research impact.

### Data set and research methodology

**Figure 1:**
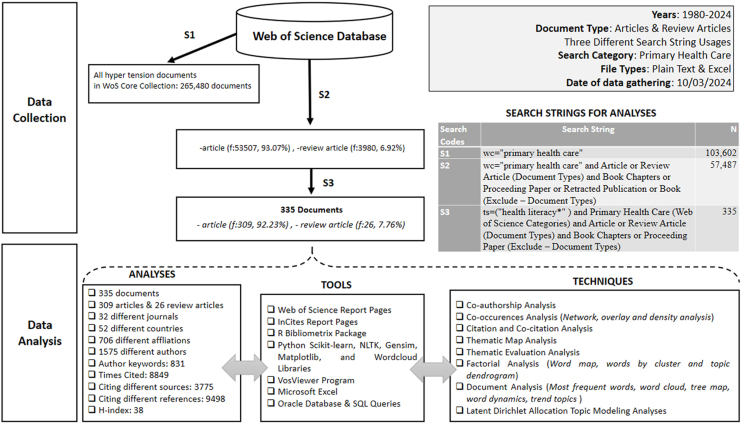
Research methodology and study design.

For our study, we selected the WoS Core Collection as the data source. We examined articles in the research domain of primary health care that contained the term “health literacy” in either the title or researcher keywords, focusing on research and review article types. Additionally, we utilized various reporting tools from WoS and Incites at different stages of the analysis. Data collection was completed on July 27, 2025. The data was obtained by following a filtering process, as outlined in Fig. 1, and the dataset relevant to query number four was extracted in the final step.

Detailed information about the analyzed data, along with explanations about the tools and techniques used, is presented in Fig. 1. The tools used for analysis included Microsoft Office Excel, Oracle Database, R Bibliometrix Biblioshiny, and VOSviewer software. Our analyses were conducted using data obtained from the WoS bibliometric data source in both Plain Text and Excel formats.

### Latent Dirichlet allocation topic modeling

Our study explores hidden patterns in the bibliometric data of 393 articles in the field of health literacy in primary healthcare services. Additionally, by revealing complex structures based on large amounts of data, the study provides researchers with a general perspective on health literacy to enable in-depth analysis of health literacy and to offer comprehensive evaluations regarding the ongoing discussions and prominent topic headings.

The dataset underwent preprocessing before topic modeling using LDA. This preprocessing stage was conducted using Python. Firstly, the text was converted to lowercase, and punctuation marks and numbers were removed. Tokenization, using the NLTK library, was applied to split the text into words, and lemmatization was performed to group similar meaningful words together. Additionally, stop words, which have no significant meaning, were removed from the text. As a result, the dataset was prepared to be suitable for topic modeling analysis using LDA.

After completing the data preprocessing stage, the dataset was divided into training and validation subsets using the scikit-learn library (a machine learning library) in Python. Then, the LDA model was applied to perform topic modeling. For visualization, the Matplotlib and Wordcloud libraries were utilized. To measure the success of the LDA method in topic modeling, the Perplexity and Coherence Score metrics were used.^21^, ^22^ These metrics are commonly preferred for evaluating LDA performance. In our analysis, 30% of the data was used for training and 70% for testing. The Perplexity metric yielded a value of 947.474, and the coherence score was 0.797.

## Results

### General view of bibliometric analyses

In our study, we analyze the scientific productivity in the field of health literacy within primary healthcare research between 1980 and 2025. A total of 393 articles, i.e., 367 research papers and 26 literature reviews, were examined. The first article in the PHC field was published in 2002. Overall, an increasing interest in the topic of health literacy has been noted over the years, with an annual growth rate of 13.91%. Of the 393 articles, 281 articles (71.50%) were published in open access journals, while 280 articles (71.24%) were indexed in the Science Citation Index Expanded (SCI-Expanded), 154 articles (39.18%) in the Social Sciences Citation Index (SSCI), and 113 articles (28.75%) in the Emerging Sources Citation Index (ESCI). It was observed that 40.71% of the publications received no institutional support. The institutions providing the most support to the research included the United States Department of Health Human Services (*n*:30, 7.63%), National Institutes of Health USA (*n*:22, 5.59%), Australian Government (*n*:8, 2.03%), National Health Medical Research Council of Australia (*n*:8, 2.03%), National Institutes of Health Research (*n*:8, 2.03%), Agency for Healthcare Research Quality (*n*:6, 2.03%), and National Health and Medical Research Council of Australia (*n*:6, 1.79%). The 393 articles were published in 33 different journals by 823 different institutions across 54 countries, involving 1869 researchers.

The top ten most productive countries in health literacy within PHC are as follows: USA (Total Cited (TC):7153, H-Index (HI):32, Average Citation Per Article (ACPA):63.87, Article Count (N):112, 28.49%), Australia (TC:1533, HI:20, ACPA:19.41, *N*:79, 20.10%), England (TC:646, HI:13, ACPA:21.53, *N*:30, 7.63%), Canada (TC:375, HI:11, ACPA:20.83, *N*:18, 4.58%), India (TC:102, HI:6, ACPA:6.93, *N*:15, 3.81%), Netherlands (TC:113, HI:6, ACPA:8.07, *N*:14, 3.56%), New Zealand (TC:144, HI:7, ACPA:10.36, *N*:14, 3.56%), South Africa (TC:113, HI:6, ACPA: 8.69, *N*:13, 3.30%), Iran (TC:205, HI:5, ACPA:17.08, *N*:12, 3.05%), and Germany (TC:115, HI:6, ACPA:10.45, *N*:11, 2.79%). Prominent researchers in health literacy within the PHC field are listed in Appendix 1, while leading institutions and countries are presented in Appendix 2.

Our analysis revealed that the 393 articles received a total of 10,900 citations, with an average citation per article of 27.74 and an H-Index of 42. To further examine the distribution of these articles, Bradford's Law was applied. According to this law, the journals occupying the first zone in terms of health literacy publications are as follows: Australian Journal of Primary Health, BMC Primary Care, Journal of Primary Care and Community Health, Journal of the American Board of Family Medicine. The journals in the second zone include: BMC Family Practice, Journal of Family Medicine and Primary Care, Family Medicine, Family Practice, Primary Care Diabetes, and African Journal of Primary Health Care & Family Medicine.

The most cited journals in the 393 articles on health literacy are: Journal of General Internal Medicine (f:367), Patient Education and Counseling (f:346), BMC Public Health (f:210), JAMA journal of the American Medical Association (f:196), Diabetes Care (f:168), PLOS One (f:146), BMC Family Practice (f:143), BMJ Open (f:140), BMJ: British Medical Journal (f:138), BMC Health Services Research (f:136).

The top 20 journals where articles on health literacy in the PHC research field are most frequently published listed in Appendix 3, and the top 20 most cited articles on health literacy in this field are provided in Appendix 4. It is noteworthy that 7 journals in Appendix 3 are indexed in ESCI, while others are indexed in SCI-Expanded (SCIE). The top five prominent journals in health literacy collectively account for approximately half of the publications in this field. Additionally, the distribution of the top 20 most cited articles across journals is as follows: Annals of Family Medicine (f:6), American Family Physician (f:3), Family Medicine (f:2), Journal of The American Board of Family Medicine (f:2), BMC Family Practice (f:2), Family Medicine and Community Health (f:1), British Journal of General Practice (f:1), Primary Care Diabetes (f:1), Family Practice (f:1), Australian Family Physician (f:1).

### Topic and research area analyses

**Figure 2:**
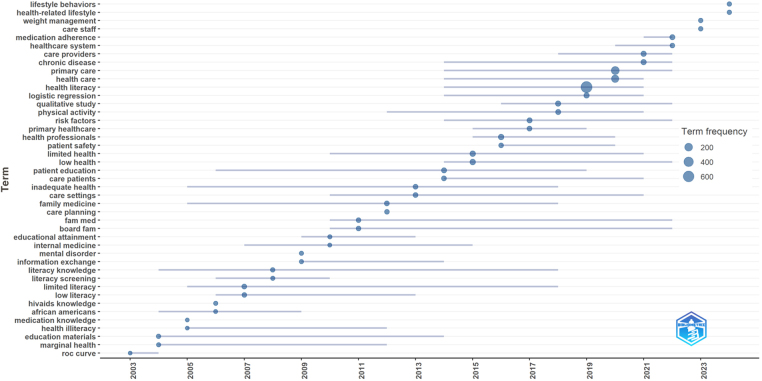
Author keywords based trend topic analyses.

**Figure 3:**
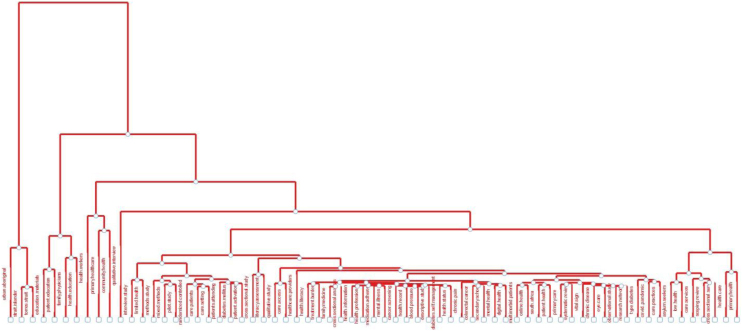
Dendrogram as a factor analysis for research titles.

Health literacy has been associated with eight different research fields besides primary healthcare research. The related fields are as follows without primary health care: Medicine general internal (f:201, 51.14%), health care sciences services (f:52, 13.23%), health policy services (f:43, 10.94%), public environmental occupational health (f:43, 10.94%), endocrinology metabolism (f:16, 4.07%), respiratory system (f:4, 1.01%), orthopedics (f:3, 0.76%), sport sciences (f:3, 0.76%). Additionally, text mining analyses were conducted based on the author's keywords, keyword plus, article titles, and abstracts. The trend topic analysis based on article author keywords is illustrated in Fig. 2, and a dendrogram, representing a factor analysis of the author's keywords, is shown in Fig. 3.

**Figure 4:**
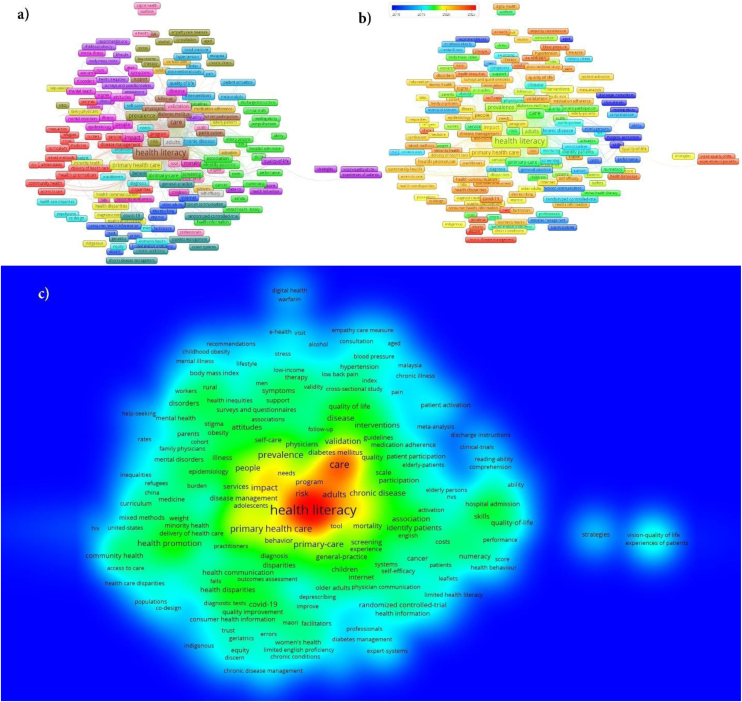
Co-occurrence analyses for authors’ keywords (a. network analyses, b. overlay analyses c. density analyses).

Fig. 4 illustrates the co-occurrence analyses for authors’ keywords, encompassing clustering of researcher keywords, network analyses, changes in researcher keywords over the years in overlay analyses, and the density representation of these keywords. The sizes of the text and text boxes in Fig. 4a–c are proportional to the frequency of the keywords. In Fig. 4a, words of the same color represent those within the same cluster, with a total of ten clusters identified. A similar color distribution can also be observed in Fig. 4c.

**Figure 5:**
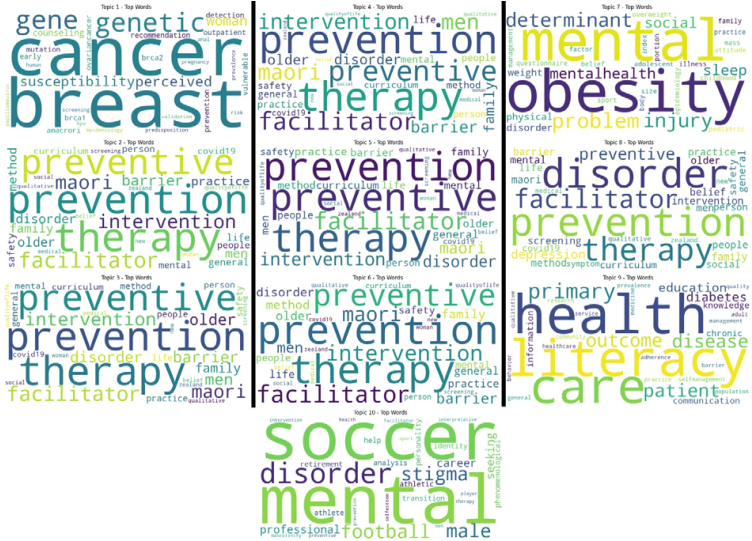
LDA analyses topic modeling for article keywords and keywords plus.

Following the combination of researcher keywords and keywords plus keywords, ten topics were identified through LDA analyses. These topics are presented in Fig. 5.

## Discussion

Numerous bibliometric studies on health literacy have been conducted, often focusing on specific conditions such as chronic diseases^23^, ^24^, ^25^, ^26^ or evaluating the topic broadly.^11^, ^27^ However, detailed research specifically focusing on primary healthcare services is lacking. In our study, instead of standard bibliometric techniques, we employed unsupervised machine learning (LDA topic modeling) on the keywords, titles, and abstracts of the articles. This approach allowed us to identify different topic clusters, which we discuss below.

### Health literacy education in schools for health equity and sustainability

Promoting health literacy at the population level enhances health equity and sustainability,^17^ influences how children and families engage with medical care and health services, and impacts overall health outcomes.^28^ Limited health literacy is linked to medication errors and poor adherence, particularly in pediatric care.^28^ Health literacy competencies should be addressed in schools to improve students’ outcomes.^29^, ^30^ The World Health Organization's European Region advocates for early health literacy education,^29^ and previous studies on children and adolescents highlight its positive impact on health behaviors.^31^ Adolescence, a critical period for developing health literacy, often sees the emergence of risk-taking behaviors and low participation in primary health services. School-based health services can improve access and engagement.^32^ Health literacy education in schools can lead to healthier societies and lifelong benefits, making schools an ideal starting point for this endeavor.^33^

### The impact of health literacy on medication use

Health literacy is crucial for medication adherence.^34^ Lower health literacy in older adults leads to insufficient medication knowledge.^35^ This highlights the vulnerability of certain age groups in healthcare. Health literacy should be considered significantly critical for such patients. Weiss et al.^36^ indicated that health literacy can also help identify medication therapy problems. Additionally, Rowlands et al.^27^ stated that complex health materials pose barriers to health. Effective strategies identified include enhancing population skills, improving healthcare providers’ communication, and enhancing written health information. Thus, leveraging health literacy in the healthcare system, starting from the primary care level, is crucial. Avazeh et al.^34^ suggested that improving access to the internet and information technologies, along with developing patient education approaches and educational techniques, are necessary to enhance patients’ health literacy. They highlighted the significant role of health policymakers in this regard.

### Health literacy in older adults

Elderly patients with inadequate health literacy struggle to actively engage in healthcare services,^37^ leading to higher rates of disease and mortality.^38^ Poor health literacy is particularly prevalent among the elderly and is associated with worse outcomes.^4^ Improving health literacy in this demographic can positively impact hospital stays, chronic disease management, and treatment adherence.^39^ Nutrition education interventions are more effective among the elderly when health literacy levels are adequate.^40^ Policymakers must develop strategies aimed at enhancing health literacy among the elderly, particularly in primary care settings. This is because health literacy is a strategic element, and the elderly population is the most vulnerable and sensitive to illnesses. The positive impact in this regard would also improve overall health outcomes and reduce healthcare costs.

### Role of health literacy in managing physical activity, dietary habits, and chronic diseases

Low health literacy is linked to poor health outcomes and longer hospital stays, particularly among those with chronic conditions.^12^ Adequate health literacy improves adherence to dietary recommendations and physical activity.^41^, ^42^, ^43^ Self-management, the cornerstone of chronic disease management, is hindered by low health literacy.^44^ Patient-centered strategies, such as reducing medical jargon and using multimedia presentations, have been effective in improving outcomes for chronic conditions.^23^ Given the role of primary healthcare services in addressing obesity, physical inactivity, and chronic diseases, strategic approaches utilizing health literacy and digital technologies such as mobile applications are needed to enhance disease management.

### Primary care health literacy, physician communication, and sustainable multidimensional health literacy

Dynamic and personalized health literacy interventions support continuous improvement in patient care.^45^ In summary, health literacy should be dynamic and transformative, and it can even be emphasized as personalized because every individual's health status may vary. Sometimes people may face rare diseases and struggle with them, where adherence to healthcare provider instructions is crucial in managing these conditions. Therefore, the patient's health knowledge and adherence to the treatment process are considered critical for physicians to monitor. Coleman and Appy^46^ stated that many medical schools in the United States include health literacy in their mandatory curricula. The content of these trainings should consider patient characteristics as a critical factor. It has been noted in the literature that various socio-demographic characteristics of patients, such as age, gender, nationality, belonging to a community, and education level, should be attended to by healthcare providers.^37^, ^38^, ^47^, ^48^

A similar situation can also apply to physicians. Sometimes physicians may encounter patients who require very specific care and communication. Ellender et al.^23^ expressed the need for reliable and practical tools for clinicians to assess health literacy. Patients understand medical information better when spoken to slowly, simple words are used, and a limited amount of information is presented. All patients prefer to read medical information written in clear and concise language. Physicians need to be cautious about this issue because most patients may not want to acknowledge literacy problems.^49^ Effective communication is crucial for patient understanding and adherence, especially among those with low health literacy.

Primary care physicians should be trained in specific skills to manage complex treatment processes and improve patient outcomes.^50^ Patients understand medical information better when it is communicated clearly and concisely. Physicians need to be aware of patients’ literacy levels to tailor education and enhance adherence.^36^ Additionally, the literature indicates that complex treatment processes also affect the level of care for patients.^3^, ^27^ Millard et al.^51^ stated that many adult patients consulting primary care physicians experience memory problems. The risky aspect is that most primary care physicians and nurses perceive their knowledge of dementia as inadequate. This situation can be encountered at every stage of the healthcare system. Moreover, it is not limited to dementia but also applies to various situations such as medication use,^36^ diabetes,^26^ hypertension,^52^ and mental illness.^53^ Creating guideline resources and supporting services of doctors and other healthcare professionals in primary care with decision support tools may be the most ideal way to address such situations.

### Socio-demographic characteristics in health literacy education

Health literacy varies by gender, language, and socio-economic status.^38^, ^47^ Culturally appropriate healthcare delivery is essential for minority and immigrant populations.^48^, ^54^ Therefore, countries with a significant immigrant population, such as Canada, the United States, and Australia, should pay more attention to this issue. Activities prioritizing health literacy can be carried out starting from primary healthcare services. Altin and Stock^54^ stated that German adults with adequate health literacy and positive experiences with care coordination and access to care were more satisfied with the care they received from their personal primary care physicians. This finding is crucial for primary healthcare institutions that aim to transform service processes to provide more effective services to their patients.

Studies in the literature also highlight the importance of who provides health literacy education or information related to healthcare. For example, low health literacy among Pacific populations affects their relationships with healthcare professionals.^55^ Somali women prefer female service providers for physical examinations.^56^ Indigenous communities often prefer storytelling and local cultural elements for health education.^57^ Provider characteristics significantly impact health literacy education, and primary healthcare institutions must transform service processes to cater to these needs effectively.

### Health literacy in rural areas

Limited health literacy is more common in socioeconomically disadvantaged rural areas.^24^, ^25^ Schools play a crucial role in promoting health literacy and healthy behaviors.^58^ Access challenges to health literacy in rural communities include limited access to healthcare services, restricted resources, low literacy rates, cultural and language barriers, financial constraints, and digital divide.^59^ Policymakers must use all available means to ensure rural populations receive adequate health literacy education and healthcare access.^60^ Socioeconomic deprivation influences healthcare decisions and referrals within the system.^61^ Schools are often suggested as ideal environments for promoting healthy habits. Health literacy offers a potential solution for encouraging such healthy behaviors.^25^ Therefore, implementing health literacy education in rural schools at an early age can lead to healthier lives and help reduce health disparities.

### Health literacy during pandemics

The COVID-19 pandemic highlighted the importance of health literacy in combating misinformation and vaccine hesitancy.^62^ Health literacy is crucial for overcoming societal perceptions during outbreaks and addressing negative attitudes toward diseases among both the public and healthcare professionals.^63^ Social media and internet broadcasts have become essential tools for public health communication during pandemics. As evidenced by the recent COVID-19 global pandemic, the world has become smaller, and misinformation during such outbreaks can lead to loss of life or endanger people's health. Therefore, promoting health literacy, particularly with regard to internet content and social media, through accredited websites from recognized authorities is deemed highly beneficial.

### Health literacy for healthcare institutions, politicians, and health advocates

Health literacy tools are vital for healthcare providers and policymakers.^38^ Collaborative efforts are needed to develop health literacy initiatives.^6^ Shared responsibility in healthcare is crucial for effective health literacy.^55^ Health literacy should be seen as a public policy issue, impacting the quality, cost, and safety of care.^59^ Various remedial activities, such as low-literacy informational brochures and valid assessment tools, have been developed to improve health literacy.^52^, ^64^ Various application examples can be utilized in primary healthcare services. As a technological option, particularly in terms of institutional mobile applications, mobile apps may be especially preferred due to their ease of access and ubiquity.

### Tools and considerations for developing health literacy materials

Health information is a crucial factor in improving individuals’ health behaviors. However, the quality and type of materials prepared at this point are of critical importance. Health information materials must be clear, concise, and accessible to improve health behaviors.^28^, ^65^ Patient education materials should ideally be written at a sixth-grade reading level or lower and include illustrations for better comprehension.^49^ Additionally, identifying patients’ health literacy levels helps clinicians personalize patient education, which in turn enhances patients’ adherence to medication regimens and their knowledge of pain management.^66^, ^67^ The emergence of digital media has provided unprecedented access to health information, but it has also brought new challenges. Managing the volume of existing information and evaluating its quality and reliability emerge as significant problems.^68^ The quality and reliability of digital health information are crucial for effective health literacy.^68^, ^69^ Online video technology and social media are effective tools for promoting health literacy.^70^, ^71^ High-quality, evidence-based materials should be made available through a centralized structure to ensure accessibility and accuracy. Emerging technologies such as augmented reality and virtual reality have significant potential for future health literacy applications.^72^

## Conclusion

Our research highlights that health literacy is a multifaceted aspect that, when well-developed, enables individuals to lead healthier lives, manage diseases effectively, prevent chronic conditions, and enhance quality of life and satisfaction with health services. Health literacy should be nurtured from a young age and integrated into lifelong learning. The COVID-19 pandemic underscored the dangers of misinformation, emphasizing the need for authoritative health literacy initiatives, especially regarding internet content. It is crucial to promote health information from credible sources and healthcare professionals, with government authorities taking preventive measures against misinformation. The importance of health literacy, effective communication, and the professional knowledge of healthcare providers is paramount. Primary healthcare services play a vital role in fostering health literacy through preventive care, early intervention, cancer screenings, diabetes management, and promoting physical activity. Policymakers should prioritize health literacy within primary healthcare services and develop mobile-supported personalized health literacy applications. Centralized, evidence-based systems accessible to all are critical for improving health literacy. Developing freely accessible web portals and mobile applications can significantly enhance health literacy, particularly in remote rural areas.

## Disclaimer

Authors hold sole responsibility for the views expressed in the manuscript, which may not necessarily reflect the opinion or policy of the Atención Primaria.

## Author contributions

Research idea: Muhammet Damar, Andrew David Pinto.

Design of the study: Muhammet Damar, Andrew David Pinto.

Acquisition of data for the study: Muhammet Damar, Ömer Aydın, Fatih Safa Erenay, Ümit Çalı.

Analysis of data for the study: Muhammet Damar, Andrew David Pinto.

Interpretation of data for the study: Muhammet Damar, Andrew David Pinto, Ömer Aydın, Fatih Safa Erenay.

Drafting the manuscript: Muhammet Damar, Andrew David Pinto, Ümit Çalı, Thiago Gomes da Trindade.

Revising it critically for important intellectual content: Muhammet Damar, Andrew David Pinto, Benita Hosseini, Thiago Gomes da Trindade.

Final approval of the version to be published: Muhammet Damar, Andrew David Pinto, Benita Hosseini, Ümit Çalı, Ömer Aydın, Fatih Safa Erenay, Thiago Gomes da Trindade.

## Ethics statement

In this study, there was no contact with the patient or any point where ethics committee approval was required. Therefore, ethics committee permission was not obtained for the study.

## Declaration of use of generative AI

No generative AI tools were used at any stage of this study.

## Source of funding

None.

## Conflict of interest

The authors declare that they have no known competing financial interests or personal relationships that could have appeared to influence the work reported in this paper.

## Data availability statement

The data that support the findings of this study are available from the corresponding author upon reasonable request.

## Figures and Tables

**Table:** 

Rank	Researcher profiles	Affiliations	Country	ACPA	HI	TC	*N*	%
1	Harris, Mark F	University of New South Wales Sydney	Australia	40.29	5	282	7	1.78
2	Weiss, Barry D.	University of Arizona	USA	309.29	7	2165	7	1.78
3	Wallace, Lorraine S.	Ohio State University	USA	25.80	5	129	5	1.27
4	Denney-wilson E	University of Sydney	Australia	59.25	3	237	4	1.01
5	Hosseinzadeh, Hassan	University of Wollongong	Australia	32.25	4	129	4	1.01
6	Lloyd J	Johns Hopkins University	USA	8.75	2	35	4	1.01
7	Rowlands, Gill	Newcastle University	England	52.00	4	208	4	1.01
8	Stock, Stephanie	University of Cologne	Germany	13.25	4	53	4	1.01
9	Bailey, Stacy Cooper	Northwestern University	USA	1.67	2	5	3	0.76
10	Chesser, Amy K.	Wichita State University	USA	7.67	3	23	3	0.76
11	Chowdhury, Nashit	University of Calgary	Canada	30.00	2	90	3	0.76
12	Coleman, Clifford A.	Oregon Health & Science University	USA	35.67	3	107	3	0.76
13	Dennis, Sarah M.	University of Sydney	Australia	76.67	3	230	3	0.76
14	Haller, Dagmar	University of Geneva	Switzerland	28.33	3	85	3	0.76
15	Maindal, Helle Terkildsen	Aarhus University	Denmark	25.00	3	75	3	0.76
16	Ng CJ	National University of Singapore	Singapore	9.33	2	28	3	0.76
17	Protheroe, Joanne	Keele University	England	68.67	3	206	3	0.76
18	Taggart, Jane	University of New South Wales Sydney	Australia	76.67	3	230	3	0.76
19	Turin, Tanvir C	University of Calgary	Canada	30.0	2	90	3	0.76
20	Van Driel M	University of Queensland	Australia	12.00	2	36	3	0.76
21	Wolf Michael S	Northwestern University	USA	1.67	2	5	3	0.76
22	Yngman-uhlin P	Linkoping University	Sweden	3.33	2	10	3	0.76

**Table:** 

Rank	Affiliations	Country	TC	HI	ACPA	*N*	%
1	University of New South Wales Sydney	Australia	363	8	22.69	16	4.07
2	University of Queensland	Australia	185	10	11.56	16	4.07
3	University of Melbourne	Australia	157	7	10.47	15	3.81
4	Monash University	Australia	155	7	12.92	12	3.05
5	University of Sydney	Australia	99	5	9.00	11	2.79
6	University of Arizona	USA	2232	8	223.20	10	2.54
7	Harvard University	USA	247	6	27.44	9	2.29
8	University System of Ohio	USA	248	7	27.56	9	2.29
9	University of California System	USA	210	4	26.25	8	2.03
10	University of Wollongong	Australia	244	8	30.50	8	2.03
11	Harvard University Medical Affiliates	USA	88	4	12.57	7	1.78
12	Netherlands Institute for Health Services Research	Netherlands	47	3	6.71	7	1.78
13	Ohio State University	USA	200	6	28.57	7	1.78
14	University of Auckland	New Zealand	61	5	8.71	7	1.78
15	University of California San Francisco	USA	210	4	30.00	7	1.78
16	Aarhus University	Denmark	248	6	41.33	6	1.52
17	Australian National University	Australia	55	5	9.17	6	1.52
18	Brigham and Women's Hospital	USA	78	3	13.00	6	1.52
19	Flinders University South Australia	Australia	59	4	9.83	6	1.52
20	University of Calgary	Canada	123	3	20.50	6	1.52
21	University of Colorado System	USA	111	5	18.50	6	1.52

**Table:** 

Rank	Journal	5JIF	Research domain	SCIE/ESCI	ACPA	HI	*N*	%
1	Australian Journal of Primary Health	1.5	Health Care Sciences & Services; Health Policy & Services; Primary Health Care; Public, Environmental & Occupational Health	SCIE	12.26	13	43	10.94
2	BMC Primary Care	2.6	Medicine, General & Internal; Primary Health Care	SCIE	3.53	7	43	10.94
3	Journal of Primary Care and Community Health	2.7	Primary Health Care	ESCI	8.26	11	43	10.94
4	Journal of The American Board of Family Medicine	2.7	Medicine, General & Internal; Primary Health Care	SCIE	23.45	16	33	8.39
5	BMC Family Practice	3.3	Medicine, General & Internal; Primary Health Care	SCIE	29.63	17	27	6.87
6	Journal of Family Medicine and Primary Care	1.6	Primary Health Care	ESCI	6.2	6	20	5.08
7	Family Medicine	2.0	Medicine, General & Internal; Primary Health Care	SCIE	139.24	13	17	4.32
8	Family Practice	2.4	Medicine, General & Internal; Primary Health Care	SCIE	24.00	9	17	4.32
9	Primary Care Diabetes	2.5	Endocrinology & Metabolism; Primary Health Care	SCIE	24.00	9	16	4.07
10	African Journal of Primary Health Care Family Medicine	2.1	Primary Health Care	ESCI	9.69	7	13	3.30
11	Journal of Primary Health Care	1.00	Primary Health Care	ESCI	6.08	6	13	3.30
12	British Journal of General Practice	6.3	Medicine, General & Internal; Primary Health Care	SCIE	37.20	9	10	2.54
13	Family Medicine and Primary Care Review	0.5	Primary Health Care	ESCI	1.30	2	10	2.54
14	Primary Health Care Research and Development	2.4	Primary Health Care	SCIE	10.30	7	10	2.54
15	Australian Journal of General Practice	2.2	Medicine, General & Internal; Primary Health Care	SCIE	4.67	5	9	2.29
16	Scandinavian Journal of Primary Health Care	2.3	Health Care Sciences & Services; Medicine, General & Internal; Primary Health Care	SCIE	9.00	6	9	2.29
17	Annals of Family Medicine	5.1	Medicine, General & Internal; Primary Health Care	SCIE	301.38	7	8	2.03
18	Australian Family Physician	1.177	Medicine, General & Internal; Primary Health Care	SCIE	27.14	5	7	1.78
19	Education For Primary Care	1.2	Primary Health Care	ESCI	5.33	4	6	1.52
20	Canadian Family Physician	3.1	Medicine, General & Internal; Primary Health Care	SCIE	13.00	5	5	1.27
21	American Family Physician	7.4	Medicine, General & Internal; Primary Health Care	SCIE	182.25	4	4	1.01
22	Atencion Primaria	1.9	Medicine, General & Internal; Primary Health Care	SCIE	10.00	4	4	1.01
23	BJGP Open	–	Primary Health Care	ESCI	8.00	2	3	0.76
24	European Journal of General Practice	2.8	Medicine, General & Internal; Primary Health Care	SCIE	20.67	2	3	0.76
25	Journal of Family Practice	0.6	Medicine, General & Internal; Primary Health Care	SCIE	13	3	3	0.76

**Table:** 

Rank	Title	Journal	ESCI/SCIE	JIF	Authors	Year	C
1	Quick assessment of literacy in primary care: The newest vital sign	Annals of Family Medicine	SCIE	5.1	Weiss, BD; Mays, MZ; (…); Hale, FA	2005	1596
2	Brief questions to identify patients with inadequate health literacy	Family Medicine	SCIE	1.7	Chew, LD; Bradley, KA and Boyko, EJ	2004	1557
3	Health literacy: The gap between physicians and patients	American Family Physician	SCIE	3.5	Safeer, RS and Keenan, J	2005	372
4	The Contribution of Health Literacy to Disparities in Self-Rated Health Status and Preventive Health Behaviors in Older Adults	Annals of Family Medicine	SCIE	5.1	Bennett, IM; Chen, J; (…); White, S	2009	365
5	The role of health literacy in patient-physician communication	Family Medicine	SCIE	1.7	Williams, MV; Davis, T; (…); Weiss, BD	2002	360
6	What is the meaning of health literacy? A systematic review and qualitative synthesis	Family Medicine and Community Health	ESCI	4.3	Liu, CX; Wang, D; (…); Zhang, XP	2020	338
7	Health Literacy in Primary Care Practice	American Family Physician	SCIE	3.5	Hersh, L; Salzman, B and Snyderman, D	2015	222
8	A systematic review of interventions in primary care to improve health literacy for chronic disease behavioral risk factors	BMC Family Practice	SCIE	3.2	Taggart, J; Williams, A; (…); Harris, MF	2012	175
9	A mismatch between population health literacy and the complexity of health information: an observational study	British Journal of General Practice	SCIE	5.2	Rowlands, G; Protheroe, J; (…); Rudd, R	2015	153
10	Health Information Seeking, Receipt, and Use in Diabetes Self-Management	Annals of Family Medicine	SCIE	5.1	Longo, DR; Schubert, SL; (…); Clore, JN	2010	139
11	Teach back and pictorial image educational strategies on knowledge about diabetes and medication/dietary adherence among low health literate patients with type 2 diabetes	Primary Care Diabetes	SCIE	2.3	Negarandeh, R; Mahmoodi, H; (…); Shakibazadeh, E	2013	131
12	Tokenism in patient engagement	Family Practice	SCIE	2.2	Hahn, DL; Hoffmann, AE; (…); Fagnan, LJ	2017	115
13	Disrupting the Pathways of Social Determinants of Health: Doula Support during Pregnancy and Childbirth	Journal of The American Board of Family Medicine	SCIE	2.6	Kozhimannil, KB; Vogelsang, CA; (…); Prasad, S	2016	115
14	Health Literacy Instrument in Family Medicine: The “Newest Vital Sign” Ease of Use and Correlates	Journal of The American Board of Family Medicine	SCIE	2.6	Shah, LC; West, P; (…); Savoy-Moore, RT	2010	115
15	Let's not talk about it: Suicide inquiry in primary care	Annals of Family Medicine	SCIE	5.1	Feldman, MD; Franks, P; (…); Kravitz, RL	2007	115
16	The effect of health literacy on knowledge and receipt of colorectal cancer screening: a survey study	BMC Family Practice	SCIE	3.2	Miller, DP; Brownlee, CD; (…); Pignone, MP	2007	107
17	Screening Questions to Predict Limited Health Literacy: A Cross-Sectional Study of Patients with Diabetes Mellitus	Annals of Family Medicine	SCIE	5.1	Jeppesen, KM; Coyle, JD and Miser, WF	2009	98
18	Health literacy A new concept for general practice?	Australian Family Physician	SCIE	–	Adams, RJ; Stocks, NP; (…); Hill, CL	2009	84
19	Managing Difficult Encounters: Understanding Physician, Patient, and Situational Factors	American Family Physician	SCIE	3.5	Lorenzetti, RC; Jacques, CHM; (…); Buck, J	2013	76
20	Promoting Health Literacy About Cancer Screening Among Muslim Immigrants in Canada: Perspectives of Imams on the Role They Can Play in Community	Journal of Primary Care and Community Health	ESCI	2.5	Khalid, A; Haque, S; (…); Turin, TC	2022	71

## Appendix 1 Most Productive Researchers in Health Literacy in PHC Field

*ACPA: Average Citation Per Articles, HI: H-Index, TC: Total Citation, *N*: Article Count.

## Appendix 2 Most Productive Institutions and Countries in Health Literacy in PHC Field

*ACPA: Average Citation Per Articles, HI: H-Index, TC: Total Citation, *N*: Article Count.

## Appendix 3 Top Journals with the Most Publications on Health Literacy in the PHC Research Field

*N*: Article Count, JIF: 5 Year Journal Impact Factor, HI: H-Index, SCIE: Science Citation Index-Expanded, ESCI: Emerging Sources Citation Index.

## Appendix 4 Top 20 Most Cited Articles on Health Literacy in the PHC Research Field

JIF: Journal Impact Factor for 2022 years, C: Citation, SCIE: Science Citation Index-Expanded, ESCI: Emerging Sources Citation Index.
